# Alpha6-Containing Nicotinic Acetylcholine Receptors Mediate Nicotine-Induced Structural Plasticity in Mouse and Human iPSC-Derived Dopaminergic Neurons

**DOI:** 10.3389/fphar.2018.00572

**Published:** 2018-06-01

**Authors:** Ginetta Collo, Laura Cavalleri, Michele Zoli, Uwe Maskos, Emiliangelo Ratti, Emilio Merlo Pich

**Affiliations:** ^1^Section of Pharmacology, Department of Molecular and Translational Medicine, University of Brescia, Brescia, Italy; ^2^Department of Biomedicine, University of Basel, Basel, Switzerland; ^3^Department of Biomedical, Metabolic and Neural Sciences, Center for Neuroscience and Neurotechnology, University of Modena and Reggio Emilia, Modena, Italy; ^4^Unité de Neurobiologie Intégrative des Systèmes Cholinergiques, CNRS UMR 3571, Institut Pasteur, Paris, France; ^5^Neuroscience Therapeutic Area Unit, Takeda Pharmaceuticals International Co., Cambridge, MA, United States; ^6^The Division of Brain Science, Imperial College London, London, United Kingdom; ^7^Neuroscience Therapeutic Area Unit, Takeda Pharmaceuticals International, Zurich, Switzerland

**Keywords:** neural plasticity, induced pluripotent stem cells, dopaminergic system, nicotine dependence, translational, dendrite outgrowth, alpha-conotoxins

## Abstract

Midbrain dopamine (DA) neurons are considered a critical substrate for the reinforcing and sensitizing effects of nicotine and tobacco dependence. While the role of the α4 and β2 subunit containing nicotinic acetylcholine receptors (α4β2^∗^nAChRs) in mediating nicotine effects on DA release and DA neuron activity has been widely explored, less information is available on their role in the morphological adaptation of the DA system to nicotine, eventually leading to dysfunctional behaviors observed in nicotine dependence. In particular, no information is available on the role of α6^∗^nAChRs in nicotine-induced structural plasticity in rodents and no direct evidence exists regarding the occurrence of structural plasticity in human DA neurons exposed to nicotine. To approach this problem, we used two parallel *in vitro* systems, mouse primary DA neuron cultures from E12.5 embryos and human DA neurons differentiated from induced pluripotent stem cells (iPSCs) of healthy donors, identified using TH^+^ immunoreactivity. In both systems, nicotine 1–10 μM produced a dose-dependent increase of maximal dendrite length, number of primary dendrites, and soma size when measured after 3 days in culture. These effects were blocked by pretreatments with the α6^∗^nAChR antagonists α-conotoxin MII and α-conotoxin PIA, as well as by the α4β2nAChR antagonist dihydro-β-erythroidine (DHβE) in both mouse and human DA neurons. Nicotine was also ineffective when the primary DA neurons were obtained from null mutant mice for either the α6 subunit or both the α4 and α6 subunits of nAChR. When pregnant mice were exposed to nicotine from gestational day 15, structural plasticity was also observed in the midbrain DA neurons of postnatal day 1 offspring only in wild-type mice and not in both null mutant mice. This study confirmed the critical role of α4α6^∗^nAChRs in mediating nicotine-induced structural plasticity in both mouse and human DA neurons, supporting the translational relevance of neurons differentiated from human iPSCs for pharmacological studies.

## Introduction

Nicotine is the primary psychoactive component of tobacco (Benowitz, 1988). Tobacco addiction has been related to the sensitizing and reinforcing properties of nicotine via its direct effect on the midbrain dopamine (DA) system (Nisell et al., 1996; Vezina et al., 2007; Grilli et al., 2009; Changeux, 2010), a critical component of the reward limbic circuits evolutionarily well conserved across vertebrates (Yamamoto and Vernier, 2011). Nicotine exerts its effects by binding to the neuronal nicotinic acetylcholine receptors (nAChRs), a heterogeneous group of pentameric ligand-gated ion channels consisting of a variety of combinations of α(2–10) and β(2–4) subunits (Changeux et al., 1984). nAChRs are differentially expressed according to their anatomical location in the brain and the stages of neurodevelopment (Zoli et al., 1995; Azam et al., 2007).

Midbrain DA neurons harbor most of the known nAChR subunits and subtypes in rodents (Klink et al., 2001) and humans (Graham et al., 2002). Several experiments were dedicated to identify the actual subunit combinations in functional nAChRs naturally expressed in the midbrain DA neurons, providing convincing evidence for the existence of α4α5β2, (non-α4)α6β2β3 α4α6β2β3 nAChRs and their involvement in controlling neuronal firing and DA release (Picciotto et al., 1998; Wonnacott et al., 2000; Champtiaux et al., 2003; Gotti et al., 2005, 2010; Grilli et al., 2009). Interestingly, a rich literature has confirmed the first observations that prolonged or repeated exposures to nicotine in rodents produced sensitization to nicotine of DA release and locomotor activity (Nisell et al., 1996; Ferrari et al., 2002; Vezina et al., 2007), as well as the development of nicotine self-administration and dependence (Picciotto et al., 1998; Merlo Pich et al., 1999; Watkins et al., 2000; Changeux, 2010).

A series of recent studies highlighted the critical role of functional nAChRs containing the α6 subunit (α6^∗^nAChRs, the ^∗^ asterisk means that other subunits may be present) in mediating the sensitizing and reinforcing effects of nicotine described above. The α6 subunit is preferentially expressed in DA neurons (Le Novère et al., 1996; Champtiaux et al., 2002) and plays a major role in controlling DA release in nucleus accumbens, firing rate in ventral tegmental area (VTA) DA neurons. Two pharmacological agents that block α6^∗^nAChRs, i.e., α-conotoxin MII (α-CTX MII) and α-conotoxin PIA (α-CTX PIA) (Dowell et al., 2003; Salminen et al., 2007), were used to study the role of α6^∗^nAChRs in motor behavior and reinforcement in mice (Drenan et al., 2008; Jackson et al., 2009; Exley et al., 2011). Knock-out of the α6 subunit, obtained using either antisense oligonucleotides or null mutation in transgenic mice, blocks the induction of locomotor activity and the reinforcing properties of nicotine (Le Novère et al., 1999; Champtiaux et al., 2003), while the reconstitution of the expression of the α6 subunit in DA neurons obtained by local infusions of viral vectors containing sequences of the native nAChR subunits, was able to normalize the pharmacological properties of nicotine, in particular its reinforcing effects (Maskos et al., 2005; Pons et al., 2008; Exley et al., 2011). Interestingly, a similar role was attributed also to the other principal subunit α4 (Marubio et al., 2003; Pons et al., 2008) and the complementary subunit β2 (Picciotto et al., 1998; Maskos et al., 2005), indicating in the α4β2^∗^, α4β2^∗^, and α4α6β2^∗^nAChRs the most critical functional receptors mediating nicotine effects on DA neurons (Drenan et al., 2010; Exley et al., 2011).

Since the development of behavioral sensitization and drug seeking are associated to neuroadaptive plasticity events (Russo et al., 2010), it has been proposed that nicotine could produce structural changes in DA neurons via α4α6^∗^nAChR activation. Using an *in vitro* approach, we recently showed in primary cultures of midbrain DA neurons obtained from embryonic day (E) 12.5 mouse embryos (Collo et al., 2013) that 1–10 μM nicotine produced structural plasticity by significantly increasing dendrite arborization and soma size when measured at 72 h. These effects were not observed in DA neurons obtained from transgenic mice with a null mutation (knock-out) in the α4 subunit (α4KO; Marubio et al., 2003; Collo et al., 2013) or following the pharmacologic blockade with the selective α4β2 nAChR antagonist dihydro-β-erythroidine (DHβE; Collo et al., 2013). Nicotine-induced structural plasticity was also sensitive to the blockade of the Akt-mTOR and Ras-ERK intracellular pathways (Collo et al., 2013) known to be involved in cell growth and neuronal plasticity (Jaworski et al., 2005; Hoeffer and Klann, 2010). Intriguingly, *in vivo* exposure of nicotine in pregnant mice starting at prenatal day E15.5 resulted in significant increases of soma size of DA neurons in the VTA and Substantia Nigra (SN) of newborn mice, effects that were absent in the α4 subunit null mutant mice (Collo et al., 2013), suggesting an impact on neurodevelopment. In mammals, including humans, prenatal exposure to nicotine/tobacco has been associated with reduced DA release (Kane et al., 2004; Alkam et al., 2017), increase liability to tobacco dependence and mesolimbic dysfunction in the offspring when assessed later in life, i.e., as adolescents (Levin et al., 2006; Goldschmidt et al., 2012; Müller et al., 2013). These observations suggest a possible long-term effect of the structural changes we observed in DA neurons of mice exposed to nicotine during the last gestational period (Collo et al., 2013). However, direct evidence that nicotine-induced structural plasticity is occurring also in human DA neurons is still missing.

To provide an initial translational assessment that nicotine induces structural plasticity also in human DA neurons, we used an *in vitro* model of human DA neurons differentiated from induced pluripotent stem cells (iPSCs) (Takahashi et al., 2007) according to standardized protocols (Kriks et al., 2011; Deflorio et al., 2017; Fedele et al., 2017) and a mouse primary neuronal culture model of DA neurons from the mouse embryos (Collo et al., 2013). The two models were assessed in parallel experiments. Human neurons differentiated from iPSCs are a recent acquisition for neuropharmacologic research (Inoue and Yamanaka, 2011); they have been successfully used for modeling monogenic central nervous system disorders, in particular those affecting neurodevelopment and early infantile life (Avior et al., 2016; Ardhanareeswaran et al., 2017) as well as neurodegenerative disorders (Cooper et al., 2012; Burbulla et al., 2017). However, relatively fewer studies were dedicated to investigate their response in pharmacological tests (Cooper et al., 2012; Oni et al., 2016; Deflorio et al., 2017; Wang et al., 2018). We recently standardized a methodology to efficiently differentiate human iPSC into DA neurons and assess the effects of pharmacologic agents on structural neuroplasticity and intracellular signaling using morphological and biochemical techniques (Fedele et al., 2017; Collo et al., 2018). In particular, in Collo et al. (2018), we studied the variability and reproducibility in iPSCs clones derived from healthy donors.

In the present work, we focused on the relevance of α6^∗^nAChRs in mediating structural plasticity produced by nicotine using pharmacological agents in mouse primary DA neuron and, for the first time, in human DA neurons differentiated from iPSCs. Additional evidence was obtained *in vivo* by exposing α6KO mice to nicotine during the sensitive late gestational period (Alkam et al., 2013).

## Materials and Methods

### Animals

The C57BL6/J mice were provided by Charles River Laboratories (Calco, Italy). The following KO mice were used: mice genetically deprived of the α6 subunit of nAChR (α6KO) or of both the α4 and α6 subunits of nAChR (α4/α6KO) (Champtiaux et al., 2002; Exley et al., 2011). Syngenic wild-type C57BL6/J mice were used as control. Animal care was in accordance with the European Community Council Directive of September 2010 (2010/63/EU) with the approval of the Institutional Animal Care and Use Committee of the University of Brescia, and in line with the Italian law. Mouse breeding was performed to achieve timed pregnancy with the accuracy of ± 0.5 days. The E was determined by considering the day of insemination (determined by vaginal plug) as day E0.5.

### Pharmacological Agents

The (-)-nicotine ditartrate (Tocris Bioscience, Bristol, United Kingdom), brain-derived neurotrophic factor (BDNF) (Alomone Labs Ltd., Jerusalem, Israel) and nAChR antagonists α-conotoxin MII (α-CTX MII), α-conotoxin PIA (α-CTX PIA), mecamylamine (MEC), dihydro-β-erythroidine (DHβE), and methyllycaconitine (MLA) (Tocris Bioscience) were used in the present study and are detailed in Supplementary Table S1. For each vehicle treatment, solvents required by specific drugs were used at the same dilution used for the active treatment.

### Mouse Primary Mesencephalic Cultures

Primary mesencephalic cultures were prepared as previously described (Collo et al., 2013). Ventral mesencephalic tissues were dissected from E12.5 C57BL6/J, α6KO, or α4/α6KO mouse embryos and mechanically dissociated in Accumax (Sigma-Aldrich, Milan, Italy). Cells were counted and seeded on poly-D-lysine/laminin (Sigma-Aldrich)-coated coverslides (5⋅10^4^/ml) in Neurobasal medium (Gibco-Invitrogen, Carlsbad, CA, United States) with the addition of 2 mM glutamine (EuroClone, Milan, Italy) and B27 supplement (Gibco-Invitrogen). Cultures were maintained at 37°C in a humidified atmosphere of 5% CO_2_ and 95% air. Pharmacological treatments were conducted at least 5 days after seeding. At this time, tyrosine hydroxylase (TH)^+^/MAP2^+^ DA neurons were about the 7–10%, of the total MAP2^+^ neurons, as previously reported (Collo et al., 2013). Further characterization of TH^+^ DA neurons was performed by double staining with dopamine transporter (DAT) (Cavalleri et al., 2017). All media and reagents are detailed in Supplementary Table S4.

### *In Vivo* Prenatal Nicotine Treatment

Pregnant α6KO, α4/α6KO, and wild-type mice were individually housed in a climate-controlled room on a 12/12-h light/dark cycle with *ad libitum* access to food and water. Treatments were performed during the light period (9:00 am – 9:00 pm) in 2–3 pregnant female per group. All mice were weighed daily starting at day 10. At day 12.5, pregnant mice weighting 25–30 g were administered intraperitoneally (i.p.) with two daily doses of 5 mg/Kg nicotine or saline as described by Collo et al. (2013). Treatments were repeated for 5 days, between E12.5 and E17.5, a time-window known to be associated with long-term behavioral effects in mice (Alkam et al., 2013). Following each injection, pregnant mice were placed for 30 min in a clean cage and inspected for locomotor activity. After delivery, P1 newborn mice (sex unknown) from at least two litters per group were sacrificed and the brains were removed and processed for immunohistochemistry with anti-TH antibody.

### Differentiation of Human iPSCs Into Midbrain DA Neuron Phenotype

Human iPSCs from the F3 clone, previously characterized (Collo et al., 2018), were induced to differentiate into floor plate (FP)-derived midbrain DA neurons using dual-SMAD inhibition and FP induction (Kriks et al., 2011; Fedele et al., 2017) with minor modifications (Collo et al., 2018). Human iPSCs were dissociated with Accutase^TM^ (StemCell Technologies, Vancouver, BC, Canada), seeded (3 × 10^4^ cells/cm^2^) on Matrigel-coated plates in Knockout Serum Replacement (KSR) medium containing Knockout^TM^ DMEM, 15% KSR, GlutaMAX^TM^, and 10 μM 2-mercaptoethanol, in the presence of LDN193189 (0.1 μM, Stemgent, Cambridge, MA, United States), SB431542 (10 μM, Tocris Bioscience), Shh C25II (0.1 μg/ml, R&D Systems), Purmorphamine (2 μM, Stemgent), Fibroblast Growth Factor 8 (0.1 μg/ml, R&D Systems), and CHIR99021 (3 μM, Stemgent). From day 5, KSR medium was gradually shifted to N2 medium (Knockout^TM^ DMEM/F12, N2 supplement, and GlutaMAX^TM^, all from Gibco-Invitrogen). On day 11, cells were passaged and replated onto fresh Matrigel coated plates at a density of 75 × 10^3^ cells/cm^2^, this procedure was repeated every 3–4 days up to 4 passages in order to expand the DA neuron-specific progenitor cell population (Fedele et al., 2017). The medium was changed to Neurobasal/B27/GlutaMAX^TM^ supplemented with CHIR99021, BDNF (20 ng/ml, R&D Systems), ascorbic acid (AA; 0.2 mM, Sigma-Aldrich), dibutyryl cAMP (cAMP; 0.5 mM, Sigma-Aldrich), transforming growth factor type β3 (TGFβ3; 1 ng/ml, R&D Systems), glial cell line-derived neurotrophic factor (GDNF; 20 ng/ml, R&D Systems), and DAPT (10 nM, Tocris Bioscience). On day 21, cells were seeded on plates pre-coated with Polyornithine/Fibronectin/Laminin and co-cultured with mouse primary cortical astrocytes (Collo et al., 2018) that were isolated and cultured according to Sorg and Magistretti (1991). Human iPSC-derived DA neurons were used for pharmacological studies, immunofluorescence, immunocytochemistry, and morphological analysis starting from day 70. A total of 3 days before pharmacological treatments, BDNF, AA, cAMP, TGFβ3, GDNF, and DAPT were gradually removed from the culture. Criteria to define DA neurons at day 70 were: neuronal morphology; co-expression of TH/MAP2; co-expression of TH/DAT; co-expression of TH/Vesicle monoamine associated transporter 2 (VMAT2); co-expression of TH/AMPAR subunit GluR2; and functional DA release and uptake from cultures containing DA neurons (Collo et al., 2018). At this time, TH^+^/MAP2^+^ DA neurons were about the 30–40%, of the total MAP2^+^ neurons, in line with previous studies (Deflorio et al., 2017; Fedele et al., 2017; Collo et al., 2018). In addition, our cultures contained VGLUT2^+^/MAP2^+^ neurons (25–30%) and GAD67^+^/MAP2^+^ neurons (20–25%) (Cavalleri et al., 2017; Collo et al., 2018). All media and reagents are detailed in Supplementary Table S4.

### *In Vitro* Pharmacological Experiments

All pharmacological treatments in human DA neurons were performed at day 70–80 in culture using several batches from the iPSC F3 clone. Pharmacological treatments on mouse mesencephalic DA neurons were performed after 5–7 days in culture. Experiments with cells from KO embryos were always run in parallel with cells from wild-type embryos. For morphological studies neuronal cultures were exposed 72 h to nicotine or BDNF, the last used as positive control. Receptor antagonists were added to the cultures 20 min prior to treatment with nicotine and cultures were fixed at 72 h. Each experiment was repeated at least twice. Each treatment group was assessed in duplicate coverslides.

### Immunofluorescence and Immunocytochemistry

Immunofluorescence was performed as previously described (Collo et al., 2013, 2018). Briefly, cultures containing mouse primary DA neurons or human iPSC-derived DA neurons were fixed with 3% paraformaldehyde in 3% sucrose/PBS (20 min at RT), blocked and permeabilized with PBS, 0.2% Triton, 1% normal goat serum, 5% bovine serum albumin (BSA, Sigma-Aldrich) (30 min at RT), and incubated overnight at 4°C with the following primary antibodies (Ab): rabbit polyclonal Ab anti-TH, mouse monoclonal Ab anti-TH, rabbit polyclonal Ab anti-MAP2, and rat monoclonal Ab anti-DAT (Supplementary Table S2). Appropriate Alexa Fluor^®^ 488- and Cy^TM^3-conjugated secondary Ab (Jackson ImmunoResearch), detailed in Supplementary Table S3, were incubated 1 h at RT, followed by DAPI (Molecular Probes – Invitrogen). Each experiment was repeated at least twice. The samples were visualized using a Zeiss Axio Observer Z1 completed with ApoTome.2 (Carl Zeiss AG, Oberkochen, Germany).

For immunocytochemistry of DA neurons, we used an anti-TH rabbit polyclonal antibody (Santa Cruz Biotechnology, Santa Cruz, CA, United States) (Supplementary Table S2), followed by incubation with a biotinylated goat anti-rabbit antibody (Jackson ImmunoResearch) (Supplementary Table S3), as previously described (Collo et al., 2013). The samples were visualized with the Olympus IX51 microscope (Olympus Italia S.R.L., Milan, Italy). All reagents are detailed in Supplementary Table S4.

### Immunohistochemistry of P1 Mouse Brain Sections

Newborn mice were sacrificed at P1 and brain removed, fixed overnight in PBS 4% paraformaldehyde, cryoprotected in 20% sucrose, and rapidly frozen by immersion in isopentane on dry ice. For each brain, a complete set of coronal sections was cut through the SN and VTA at 30 μm. Sections were mounted on slides, blocked, and permeabilized with PBS, 5% BSA, 0.1% Triton for 30 min at RT. They were incubated with an anti-TH rabbit polyclonal antibody diluted in PBS, 1% BSA, overnight at 4°C, followed by a biotinylated goat anti-rabbit antibody (Jackson ImmunoResearch) diluted in PBS, 1% BSA for 30 min at RT, and by the ABComplex and development with DAB. All reagents are detailed in Supplementary Table S4.

### Computer-Assisted Morphological Analysis

Digital images were acquired with an Olympus IX51 microscope connected to an Olympus (Hamburg, Germany) digital camera and a PC. Morphometric measurements were performed by a blinded examiner on digitalized images using Image-Pro Plus software (Media Cybernetics, Bethesda, MD, United States). For the *in vitro* studies, morphological indicators of structural plasticity of DA (TH^+^) neurons were considered: (i) the maximal dendrite length, (ii) the number of primary dendrites, and (iii) the soma area (Collo et al., 2013). Maximal dendrite length was defined as the distance from the soma (hillock base) to the tip of the longest dendrite for each neuron; dendrites shorter than 20 μm were excluded from the analysis. Primary dendrites were defined as those directly stemming from the soma. Soma area was assessed by measuring the surface (μm^2^) included by the external perimeter drawn on the cell membrane of neurons identified by TH^+^ staining. Two coverslides per treatment groups were examined so to obtain measurements from at least 30–50 neurons. Each experiment was repeated 2–3 times. For *in vivo* studies, computer-assisted morphometry was performed as described in Collo et al. (2013, 2018). The effects of nicotine on newborn mice were assessed by measuring the soma area of 40–50 DA neurons of SN and VTA, respectively, in each brain. The same rostrocaudal levels were considered for each treatment group so to keep anatomical variance partially controlled. In a typical experiment, 4–5 newborn mice from three different mothers per group were included. The soma area of midbrain DA neurons on histologic brain sections was measured using the same computer assisted approach described for *in vitro* cultures of mouse primary DA neurons.

### Statistical Analysis

Data were expressed as mean ± standard error of the mean (SEM) if not stated otherwise. Significant differences from control conditions were determined using either one-way or two-way analysis of variance (ANOVA) followed by *a posteriori* Bonferroni’s test for multiple comparisons provided by GraphPad Prism, version 6.0 software package (GraphPad Software, San Diego, CA, United States). Two-way ANOVA was generally performed between the factor “nicotine/vehicle” and the factor “antagonist” which includes the various pharmacological antagonists used.

## Results

### Effect of α-Conotoxin MII on Nicotine-Induced Structural Plasticity in Primary Cultures of Mouse DA Neurons

**FIGURE 1:**
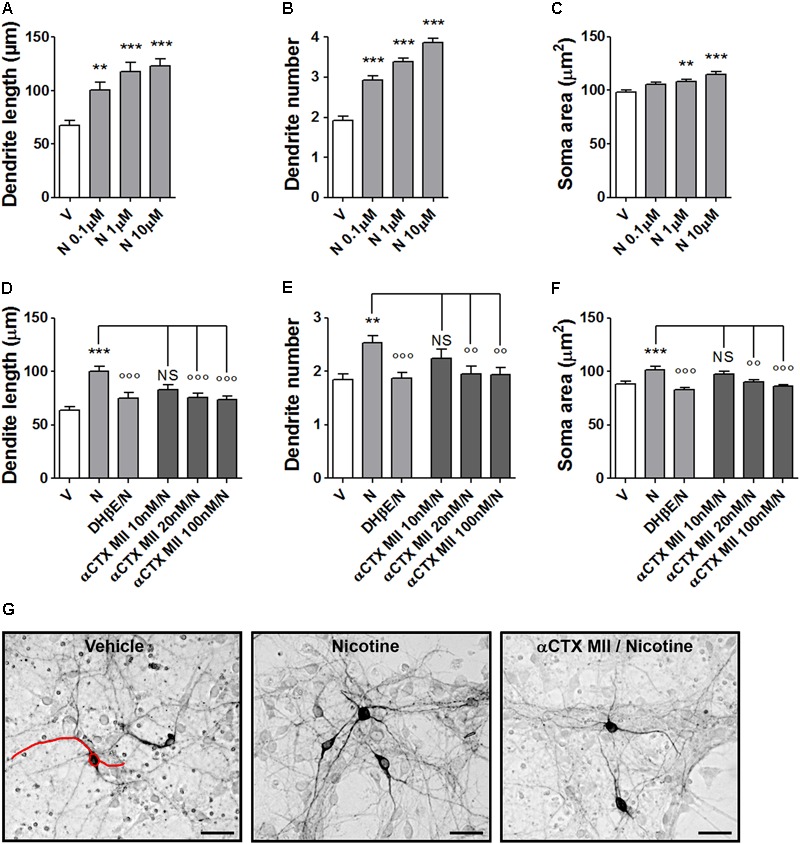
Structural plasticity induced by nicotine in mouse DA neurons and blockade with α-conotoxin MII. **(A–C)** Concentration–response curves of the effect of nicotine (0.1–10 μM) on structural plasticity measured as maximal dendrite length, number of primary dendrites, and soma area. **(D–F)** Antagonism of the 10 μM nicotine-induced structural plasticity produced by pretreatment with 10–100 nM α-conotoxin MII; 10 μM dihydro-β-erythroidine was used as internal standard; **(D)** maximal dendrite length, **(E)** number of primary dendrites, and **(F)** soma area. **(G)** Representative photomicrographs of mouse mesencephalic DA neurons 72 h after exposure to vehicle, 10 μM nicotine, or 100 nM α-conotoxin MII followed by 10 μM nicotine. Red line drawing shows how the measurements of the three parameters, dendrite length, dendrite number, and soma area were performed (Scale bar: 50 μm). One-way ANOVA was used to analyze data in panels **(A–C)** and **(D–F)**, respectively. Student’s *t*-test was used to compare dihydro-β-erythroidine with nicotine in **(D–F)**. Data are expressed as mean ± SEM (*^∗∗∗^p* < 0.001; *^∗∗^p* < 0.01 vs. vehicle; *^∘∘∘^p* < 0.001; *^∘∘^p* < 0.01 vs. nicotine; NS, non-significant, *post hoc* Bonferroni’s test). V: vehicle; N: nicotine; αCTX MII: α-conotoxin MII; DHβE: dihydro-β-erythroidine.

Nicotine produced concentration-dependent effects on morphology of DA neurons stained with an anti-TH antibody at 72 h after exposure. One-way ANOVA indicated significant treatment effects for maximal dendrite length [*F*(3,145) = 13.17; *p* < 0.0001], primary dendrite number [*F*(3,196) = 55.41; *p* < 0.0001], and soma area [*F*(3,240) = 12.33; *p* < 0.0001] (**Figures 1A–C**). Strong signal was observed with 1–10 μM, confirming previous observations (Collo et al., 2013). The involvement of α6^∗^nAChR in nicotine-induced structural plasticity of mouse DA neurons was tested by pretreatments with various concentrations of α-CTX MII on the effect produced by 10 μM nicotine (**Figures 1D–G** and Supplementary Figure S1). One-way ANOVA indicated significant treatment effects for maximal dendrite length [*F*(4,167) = 11.50; *p* < 0.0001], primary dendrite number [*F*(4,220) = 4.38; *p* < 0.002], and soma area [*F*(4,195) = 7.71; *p* < 0.0001]. All three parameters showed a significant reduction at 20 and 100 nM (Bonferroni’s test *p* < 0.01 or less), but not at 10 nM. The incubations with α-CTX MII alone was not different from vehicle (data not shown). In each experiment, DHβE (10 μM) was used as internal positive control based on previous studies (Collo et al., 2013). The DHβE antagonism of nicotine effect was significant for maximal dendrite length [*t*(66) = 3,6, *p* < 0.001], primary dendrite number [*t*(97) = 4.0, *p* < 0.001], and soma area [*t*(60) = 5.5, *p* < 0.0001]. The magnitude of the blocking effects of α-CTX MII on nicotine-induced structural plasticity was of the same order of what observed with DHβE (**Figures 1D–F**).

### Effect of α-Conotoxin PIA on Nicotine-Induced Structural Plasticity in Primary Cultures of Mouse Mesencephalic DA Neurons

**FIGURE 2:**
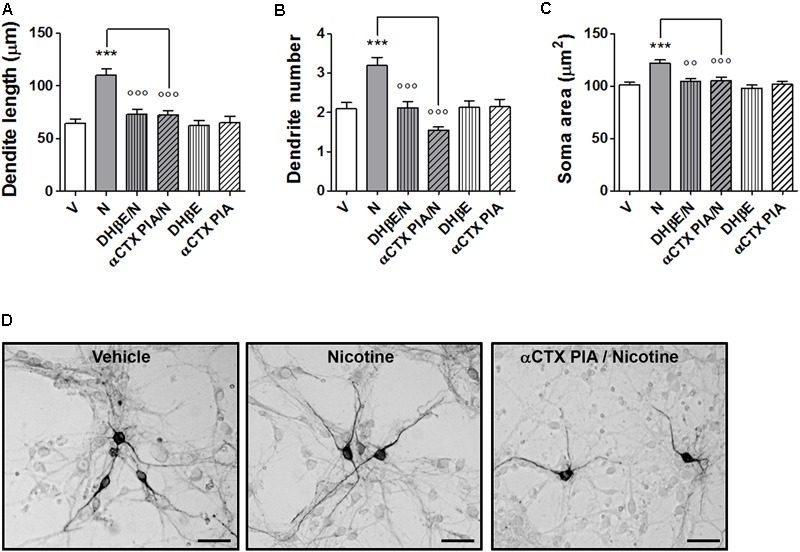
Blockade of nicotine-induced structural plasticity by α-conotoxin PIA. **(A–C)** Antagonism of the 10 μM nicotine-induced structural plasticity produced by pretreatment with 100 nM α-conotoxin PIA; 10 μM dihydro-β-erythroidine was used as internal standard; **(A)** maximal dendrite length, **(B)** number of primary dendrites, and **(C)** soma area. **(D)** Representative photomicrographs of mouse mesencephalic DA neurons 72 h after exposure to vehicle, 10 μM nicotine or 100 nM α-conotoxin PIA followed by 10 μM nicotine (Scale bar: 50 μm). Two-way ANOVA was used to analyze data in panels **(A–C)**. Data are expressed as mean ± SEM (*^∗∗∗^p* < 0.001 vs. vehicle; *^∘∘∘^p* < 0.001; *^∘∘^p* < 0.01 vs. nicotine, *post hoc* Bonferroni’s test). V: vehicle; N: nicotine; αCTX PIA: α-conotoxin PIA; DHβE: dihydro-β-erythroidine.

Pretreatments with the α6^∗^nAChR selective blocker α-CTX PIA resulted in a complete blockade of the nicotine-induced structural plasticity of mouse midbrain DA neurons (**Figures 2A–D** and Supplementary Figure S2). Two-way ANOVA of maximal dendrite length data showed a significant nicotine effect [*F*(1, 173) = 22.33, *p* < 0.0001], a significant antagonist effect [*F*(2,173) = 11,23, *p* < 0.0001], and a significant interaction [*F*(2,173) = 8.25, *p* < 0.0005]; similar results were observed for the primary dendrite number, showing a significant nicotine effect [*F*(1, 168) = 4.33, *p* < 0.05], a significant antagonist effects [*F*(2,168) = 4,03, *p* < 0.05], and a significant interaction [*F*(2,168) = 6.47, *p* < 0.002] as well as for the soma area, showing a significant nicotine effect [*F*(1, 234) = 15.89, *p* < 0.0001], a significant antagonist effect [*F*(2,234) = 8,41, *p* < 0.0005], and a significant interaction [*F*(2,234) = 7.37, *p* < 0.001]. *Post hoc* tests (**Figures 2A–C**) indicated a significant difference of 100 nM α-CTX PIA from vehicle (Bonferroni’s test *p* < 0.05 or less). DHβE (10 μM) was also blocking nicotine effects (*p* < 0.05), being used as “internal control”. When vehicle was added instead of nicotine, α-CTX PIA and DHβE did not show difference from vehicle alone.

### Structural Plasticity in Primary Cultures of Mesencephalic DA Neurons From Wild-Type Mice and α6KO or α4/α6KO nAChR Mice

**FIGURE 3:**
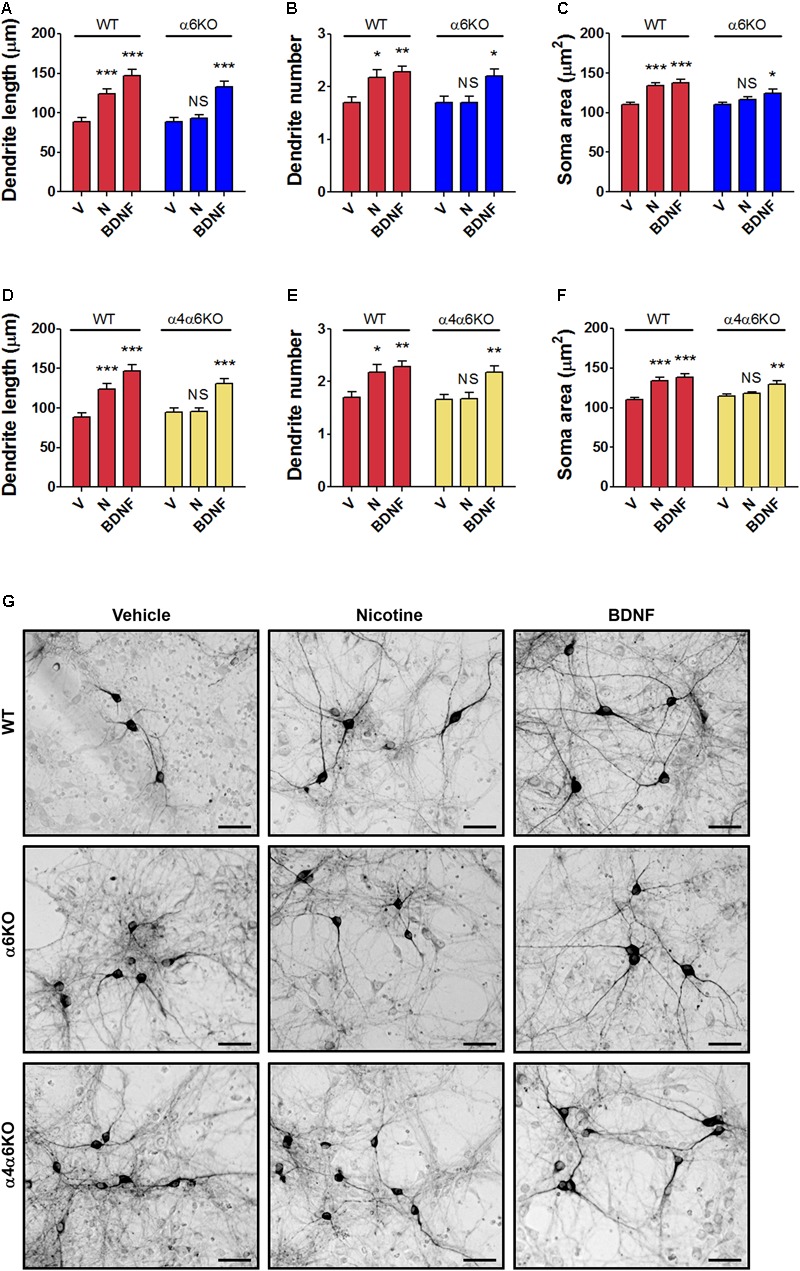
Structural plasticity induced by nicotine in mesencephalic DA neurons from wild-type (WT) mice and α6 or α4/α6 nAChR subunit null mutant mice. Morphological effects of nicotine on DA neurons from **(A–C)** α6KO and WT mice and **(D–F)** α4/α6KO and WT mice on maximal dendrite length **(A,D)**, number of primary dendrites **(B,E)** and soma area **(C,F)** measured 72 h after exposure to nicotine (10 μM), or BDNF (10 ng/ml) here used as active control group. Two-way ANOVA was used to analyze data in panels **(A–C)** and **(D–F)**, respectively. Data are expressed as mean ± SEM (*^∗∗∗^p* < 0.001; *^∗∗^p* < 0.01; *^∗^p* < 0.05 vs. vehicle; NS, non-significant, *post hoc* Bonferroni’s test). **(G)** Representative photomicrographs of mouse mesencephalic DA neurons from WT, α6KO and α4/α6KO mice 72 h after exposure to vehicle, 10 μM nicotine or 10 ng/ml BDNF (Scale bar: 50 μm). WT: wild-type, V: vehicle, N: nicotine, BDNF: brain-derived neurotrophic factor.

The structural plasticity effects of nicotine were studied in primary DA neuronal cultures of mesencephalon obtained from α6KO or from α4/α6KO mice, as well as wild-type mice (**Figures 3A–G** and Supplementary Figure S3). These KO mice were previously studied, showing a significant lack of locomotor activity and reinforcement behavior produced by nicotine (Champtiaux et al., 2002; Pons et al., 2008; Exley et al., 2011). Moreover both subunits, α4 and α6 were shown to play a major role influencing DA release in the striatum (Exley et al., 2011). In the present study, structural plasticity was measured at 72 h after treatments with either vehicle, 10 μM nicotine, or 10 ng/ml BDNF, the latter used as biological control.

Data from the experiment with α6KO mice DA neurons were analyzed using two-way ANOVA, showing for maximal dendrite length a significant Treatment effect [*F*(1,174) = 34.67, *p* < 0.0001], a significant Genotype effect [*F*(1,174) = 8.08, *p* < 0.01], and a significant interaction [*F*(2,174) = 3.17, *p* < 0.05]; for primary dendrite number, the Treatment effect was significant [*F*(2,264) = 9,77, *p* < 0.0001], but not the Genotype effect [*F*(1,264) = 3.30, NS] and interaction [*F*(2,264) = 2.20, NS]; for the soma area, the Treatment effect [*F*(2,234) = 16.75, *p* < 0.0001] and the Genotype effect [*F*(1,234) = 10.08, *p* < 0.005] were both significant, but not the interaction [*F*(1,234) = 2.82, NS] (**Figures 3A–C**).

Data from the experiment with α6KO mice DA neurons were also analyzed using two-way ANOVA, showing for maximal dendrite length a significant Treatment effect [*F*(1,174) = 30.39, *p* < 0.0001], a significant Genotype effect [*F*(1,174) = 6.76, *p* < 0.01], and a significant interaction [*F*(2,174) = 4.17, *p* < 0.05], for primary dendrite number the Treatment effect was significant [*F*(1,294) = 5.14, *p* < 0.05] and so the Genotype effects [*F*(2,294) = 11,41, *p* < 0.0001], but not the interaction [*F*(2,294) = 2.35, NS]. Finally, for the soma area the Treatment effect was significant [*F*(2,234) = 20.35, *p* < 0.0001] and so the Genotype effect [*F*(1,234) = 5.60, *p* < 0.05] and the interaction [*F*(2,234) = 4.37, *p* < 0.05] (**Figures 3D–F**).

*Post hoc* assessment (**Figures 3A–F**) of both experiments showed that cultures of DA neurons prepared from wild-type mice responded significantly to both nicotine and BDNF for all three structural plasticity parameters (Bonferroni’s test *p* < 0.01), while cultures of DA neurons prepared from the mesencephalon of α6KO or α4/α6KO mice responded only to BDNF (Bonferroni’s test *p* < 0.05), but not to nicotine. The main effect of Genotype or the interaction was not significant in some analysis, a result possibly due to a variable baseline/vehicle data obtained in the different experiments, while the main effect of Treatment was always highly significant.

### Structural Plasticity in Midbrain DA Neurons Produced in the Newborn Offspring of Wild-Type and α6KO and α4/α6KO nAChR Mice Exposed to Nicotine During Pregnancy

**FIGURE 4:**
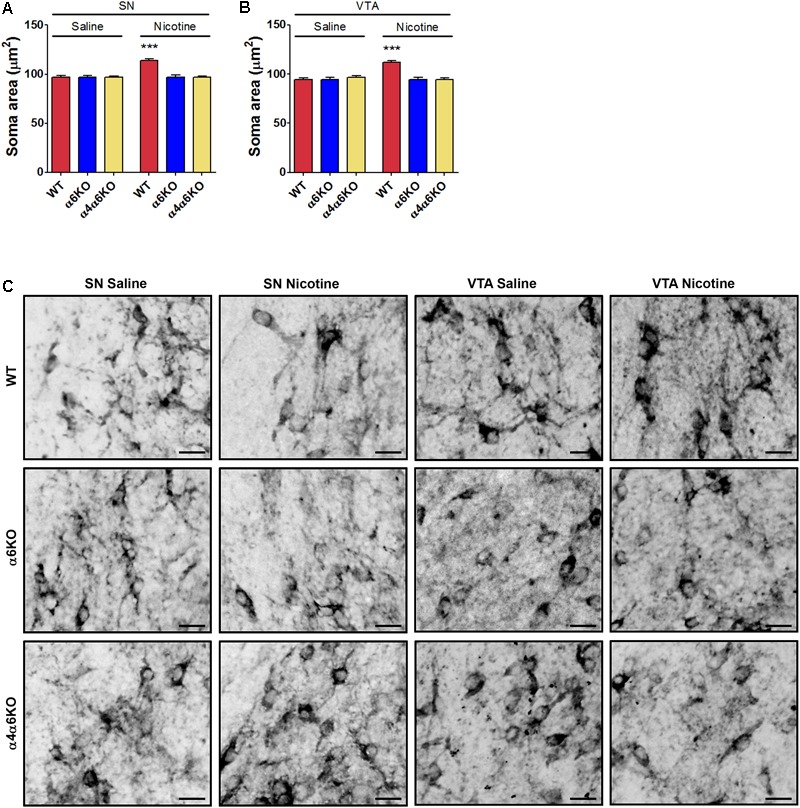
Effects of prenatal exposure to nicotine on structural plasticity of DA neurons of wild-type (WT) and α6 or α4/α6 nAChR subunit null mutant newborn mice. **(A,B)** Nicotine effects on the soma area of DA neurons in SN **(A)** and VTA **(B)** of WT, α6KO and α4/α6KO mice. Two-way ANOVA was used to analyze data in panels **(A)** and **(B)**, respectively. Data are expressed as mean ± SEM (^∗∗∗^*p* < 0.001 vs. saline; *post hoc* Bonferroni’s test). **(C)** Representative high-magnification photomicrographs of DA neurons from SN and VTA of WT, α6KO and α4/α6KO P1 mice exposed in utero to daily treatment (from E12.5 to E17.5) with saline or nicotine, i.p., (5 mg/Kg/die) (Scale bar: 30 μm). WT: wild-type; SN: substantia nigra; VTA: ventral tegmental area.

The *in vivo* relevance of the nicotine-induced changes observed *in vitro* was investigated by repeatedly exposing mouse embryos to nicotine while *in utero* from E12.5 to E17.5. Treatments were performed in parallel in wild-type and in α6KO and α4/α6KO pregnant mice. Structural plasticity was studied as changes in the soma area of midbrain TH^+^ DA neurons, i.e., in the VTA and SN, at postnatal day 1 (PND1). The focus on soma area was justified based on the difficulties in quantification of the overlapping TH^+^ dendritic branches, as previously reported (Collo et al., 2012, 2013) (**Figures 4A–C** and Supplementary Figure S4). In SN, the two-way ANOVA of soma area showed a significant Treatment effect [*F*(1,1080) = 15.87; *p* < 0.0001], a significant Genotype main effect [*F*(2,1080) = 14.13; *p* < 0.0001], and a significant interaction [*F*(2,1080) = 14.12; *p* < 0.0001], *post hoc* Bonferroni’s test indicating effect of nicotine only in wild-type mice (*p* < 0.001) (**Figure 4A**). A similar pattern was observed in VTA: two-way ANOVA of soma area showed a significant Treatment main effect [*F*(1,1063) = 12.69; *p* < 0.0001], a significant Genotype main effect [*F*(2,1063) = 14.07; *p* < 0.0001], and a significant interaction [*F*(2,1063) = 17.95; *p* < 0.0001], *post hoc* Bonferroni’s test indicating effect of nicotine only in wild-type mice (*p* < 0.001) (**Figure 4B**).

### Nicotine Induces Structural Plasticity in Human DA Neurons Differentiated From iPSCs of Healthy Donors

**FIGURE 5:**
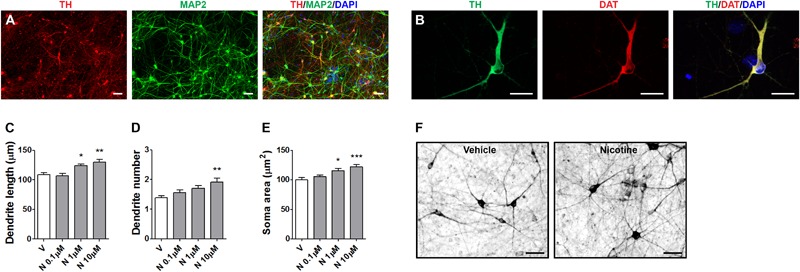
Structural plasticity induced by nicotine in human iPSC-derived DA neurons. Representative images of dual immunofluorescence indicating coexpression of **(A)** TH (red) and MAP2 (green) and **(B)** TH (green) and DAT (red) assessed at day 70. Cell nuclei were stained with DAPI (blue). Scale bar: **(B)** = 50 μm; **(C)** = 30 μm. **(C–E)** Concentration–response of nicotine effects on structural plasticity measured as **(C)** maximal dendrite length, **(D)** number of primary dendrites, **(E)** soma area. One-way ANOVA was used to analyze data in panels **(C–E)**. **(F)** Representative photomicrographs of human iPSC-derived DA neurons 72 h after exposure to vehicle or 10 μM nicotine (Scale bar: 50 μm). Data are expressed as mean ± SEM. (*^∗∗∗^p* < 0.001; *^∗∗^p* < 0.01; *^∗^p* < 0.05 vs. vehicle, *post hoc* Bonferroni’s test). V: vehicle, N: nicotine.

The DA neurons were differentiated from the human F3 iPSC clone (Collo et al., 2018) following the procedure of Fedele et al. (2017) (Supplementary Figure S5A). Midbrain FP neural precursors at day 11 showed distinctive co-expression of the FP marker FOXA2 and the roof plate marker LMX1-A (Kriks et al., 2011). Semi-quantitative RT-PCR analysis showed the expression of LMX1-A, LMX1-B, FOXA2, ENGRAILED 1 (EN1), TH, and Dopa decarboxylase (DDC), G protein-coupled inwardly rectifying potassium channel (GIRK2) starting from day 11, while NURR1 was detected from day 19 (data not shown, see Collo et al., 2018). At day 21, cells were plated on mouse astrocyte feeder layer and starting at day 30 MAP2^+^-TH^+^ neurons were present. At day 70, TH^+^ neurons co-expressed MAP2 (**Figure 5A**). At this stage, most TH^+^ neurons co-expressed DAT (**Figure 5B**). The AMPAR subunits GLUR1 and GluR2 and VMAT2 were also detected indicating a mature DA neuronal phenotype (data not shown, see Collo et al., 2018).

**FIGURE 6:**
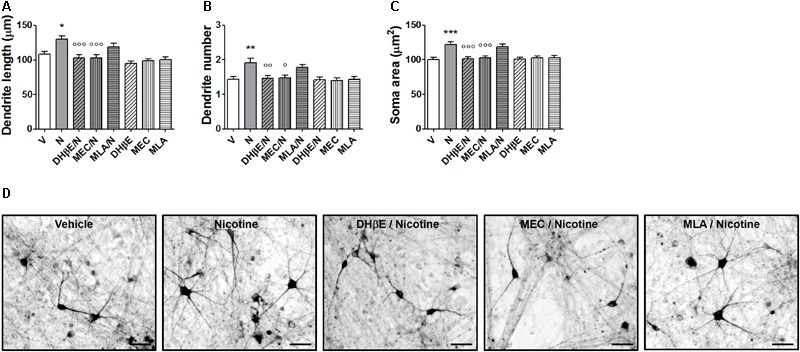
Blockade of nicotine-induced structural plasticity in DA neurons differentiated from human iPSCs by nAChR antagonists. **(A–C)** Antagonism of the 10 μM nicotine-induced structural plasticity following pretreatment with 10 μM dihydro-β-erythroidine, 100 μM mecamylamine, and 200 nM methyllycaconitine; **(A)** maximal dendrite length, **(B)** number of primary dendrites, and **(C)** soma area. **(D)** Representative photomicrographs of human DA neurons at 70 days in culture, measured at 72 h after exposure to vehicle, nicotine or pretreatments with dihydro-β-erythroidine, mecamylamine, and methyllycaconitine followed by nicotine. Two-way ANOVA was used to analyze data in panels **(A–C).** Data are expressed as mean ± SEM (*^∗∗∗^p* < 0.001; *^∗∗^p* < 0.01; *^∗^p* < 0.05 vs. vehicle; *^∘∘∘^p* < 0.001; *^∘∘^p* < 0.01; *°p* < 0.01 vs. nicotine, *post hoc* Bonferroni’s test) (Scale bar: 50 μm). V: vehicle; N: nicotine; DHβE: dihydro-β-erythroidine, MEC: mecamylamine, MLA: methyllycaconitine.

In the present experiments, human DA neurons at 70–80 days of differentiation were exposed to various doses nicotine (0.1–10 μM), producing a dose-dependent effect on structural plasticity measured on TH^+^ neurons at 72 h after the beginning of treatment (**Figures 5C–F** and Supplementary Figure S5B). One-way ANOVA indicated significant treatment effects for maximal dendrite length [*F*(3,116) = 8.37; *p* < 0.0001], primary dendrite number [*F*(3,196) = 5.64; *p* < 0.001], and soma area [*F*(3,156) = 7.7; *p* < 0.0001]. The nAChR antagonists mecamilamine, DHβE, but not MLA, blocked the effects of 10 μM nicotine (**Figures 6A–D** and Supplementary Figure S6). Two-way ANOVA for maximal dendrite length showed a significant nicotine main effect [*F*(1,232) = 19.01, *p* < 0.0001], a significant antagonist effect [*F*(3,232) = 9,19, *p* < 0.0001], and a statistically non-significant interaction [*F*(3,232) = 1.83, NS]; for primary dendrite number, a significant nicotine effect [*F*(1,392) = 13.07, *p* < 0.0002], a significant antagonist effect [*F*(3,392) = 3.68, *p* < 0.05], and a significant interaction [*F*(3,392) = 2.75, *p* < 0.05]; for soma area, a significant nicotine effect [*F*(1,312) = 18.97, *p* < 0.0001], a significant antagonist effect [*F*(3,312) = 4,99, *p* < 0.005], and a significant interaction [*F*(3,312) = 5.66, *p* < 0.001]. *Post hoc* analysis indicated a significant effect for DHβE and MEC (*p* < 0.001), but no effect was observed with the selective α7-nAChR inhibitor MLA. When vehicle was added instead of nicotine, DHβE, MEC, or MLA, no difference from vehicle was observed (**Figures 6A–C**).

### Effect of α-conotoxin MII and α-conotoxin PIA on Nicotine-Induced Structural Plasticity in Human DA Neurons Differentiated From iPSCs of Healthy Donors

**FIGURE 7:**
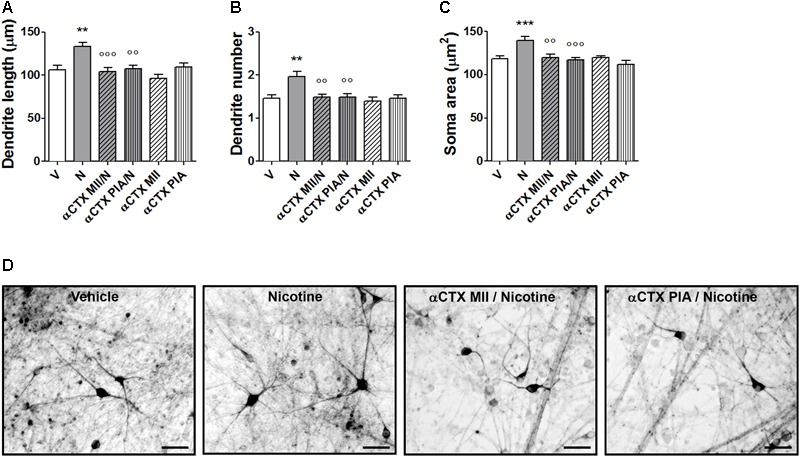
Blockade of nicotine-induced structural plasticity in DA neurons differentiated from human iPSCs by α-conotoxin MII and α-conotoxin PIA. **(A–C)** Antagonism of the 10 μM nicotine-induced structural plasticity following pretreatment with 100 nM α-conotoxin MII or 100 nM α-conotoxin PIA; **(A)** maximal dendrite length, **(B)** number of primary dendrites, and **(C)** soma area. **(D)** Representative photomicrographs of human DA neurons at 70 days in culture, measured at 72 h after exposure to vehicle, nicotine, or pretreatments with α-conotoxin MII and α-conotoxin PIA followed by nicotine. Two-way ANOVA was used to analyze data in panels **(A–C)**. Data are expressed as mean ± SEM (*^∗∗∗^p* < 0.001; *^∗∗^p* < 0.01 vs. vehicle; *^∘∘∘^p* < 0.001; *^∘∘^p* < 0.01 vs. nicotine, *post hoc* Bonferroni’s test) (Scale bar: 50 μm). V: vehicle; N: nicotine; αCTX MII: α-conotoxin MII; αCTX PIA: α-conotoxin PIA.

Pretreatments with α-CTX MII or α-CTX PIA resulted in a complete blockade of nicotine-induced structural plasticity of human DA neurons (**Figures 7A–D** and Supplementary Figure S7). Two-way ANOVA of maximal dendrite length data showed a significant nicotine effect [*F*(1,174) = 8.07, *p* < 0.005], a significant antagonist effect [*F*(2,174) = 8,73, *p* < 0.001], and a significant interaction [*F*(2,174) = 4.79, *p* < 0.01]; similar results were observed for the primary dendrite number, showing a significant nicotine effect [*F*(1,294) = 7.37, *p* < 0.01], a significant antagonist effect [*F*(2,294) = 5,38, *p* < 0.01], and a significant interaction [*F*(2,294) = 4.20, *p* < 0.05], as well as for the soma area, showing a significant nicotine effect [*F*(1,234) = 8.80, *p* < 0.001], a significant antagonist effect [*F*(2,234) = 7.92, *p* < 0.001] and a significant interaction [*F*(2,234) = 4.58, *p* < 0.05]. *Post hoc* tests indicated the significant effects of nicotine over vehicle which is significantly antagonized by both α-CTX MII and α-CTX PIA (Bonferroni’s test *p* < 0.01 or less). When vehicle was added instead of nicotine, both α-CTX MII and α-CTX PIA did not show difference from vehicle alone (**Figures 7A–C**).

## Discussion

In this article, we attempted to provide translational evidence that exposure to nicotine can induce structural plasticity in both mouse and human DA neurons through the involvement of the α6^∗^nAChR. To test this hypothesis, we used the same primary cultures of mesencephalic DA neurons from the E12.5 mouse embryo previously used to study plasticity produced by nicotine (Collo et al., 2013) and, in parallel, human DA neurons differentiated from iPSCs from healthy volunteers according to a standardized paradigm (Kriks et al., 2011; Deflorio et al., 2017; Fedele et al., 2017; Collo et al., 2018). Mouse cultures gave a yield of TH^+^/MAP2^+^ DA neurons of about 7–10% (Collo et al., 2013) while in human cultures TH^+^/MAP2^+^ DA neurons were about 30–40%, in line with previous studies (Deflorio et al., 2017; Fedele et al., 2017; Collo et al., 2018). Both cultures contained also glutamatergic (VGLUT^+^) and GABAergic (GAD67^+^) neurons. Human DA neurons differentiated from iPSCs express several nAChR subunit transcripts, including those encoding for α4, α6, α7, β2, and β3 (Deflorio et al., 2017). Measurements of dendritic length and number and of soma area performed 3 days after exposure to nicotine at the low micromolar concentration range (1–10 μM) revealed a dose-dependent effect in human DA neurons that closely reminds that seen in mouse DA neurons (Collo et al., 2013). These nicotine concentrations were compatible with the exposure reached *in vivo* and associated with behavioral effects in rodents (Changeux, 2010). Structural plasticity was blocked in both mice and human DA neurons by α-CTX MII and by α-CTX PIA, two compounds that antagonize nicotine and acetylcholine effects on α6^∗^AChRs by binding to the α6 subunit (Dowell et al., 2003; Salminen et al., 2007; Capelli et al., 2011). These results are also in line with the α-CTX MII blockade of the electrophysiological effects of nicotine observed in iPSC-derived human DA neurons (Deflorio et al., 2017). α-CTX MII is considered less selective than α-CTX PIA, since it binds with high affinity also to α3β2^∗^nAChRs (Cartier et al., 1996), that are, however, poorly expressed by midbrain DA neurons (Gotti et al., 2010). Interestingly, microinjections of α-CTX MII in the VTA were shown to attenuate reinforced behavior in rodents, an effect that was associated to functional α4α6β2β3 nAChRs identified by immunoprecipitation (Gotti et al., 2010). In the present experiment, nicotine was completely ineffective in producing structural plasticity in primary cultures of DA neurons obtained from α6 subunit null mutant mice. This lack of nicotine effect was also replicated in primary cultures of DA neurons from transgenic mice devoid of both α4 and α6 subunits. The fact that deletion of α4 subunit (Collo et al., 2013), deletion of α6 subunit, or deletion of α4 and α6 subunits together, all produced the same lack of nicotine-induced morphological effect demonstrates that both subunits are necessary to mediate nicotine-induced structural plasticity. The lack of effects of nicotine in these DA neuron preparations was not due to a non-specific impairment of the intracellular neurotrophic signaling since BDNF was able to trigger structural plasticity, as expected.

Indeed, we also previously demonstrated that nicotine activates the phosphorylation of Ras-ERK and PI3K-Akt-mTOR pathways to induce structural plasticity in DA neurons, probably by directly engaging Ca^++^ signaling. For example, enhancement of the AMPA-dependent Ca^++^ signaling by nicotine via α4α6^∗^nAChRs was shown in DA neurons (Engle et al., 2013). However, a possible indirect effect via DA release and DA D2/D3 autoreceptor activation was also proposed (Grilli et al., 2009; Collo et al., 2013). The releasing properties of nicotine on DA are well known and occur both at the synaptic level, in the rostral terminal fields, e.g., the nucleus accumbens (Picciotto et al., 1998; Grilli et al., 2009), and at the somato-dendritic level, e.g., in the VTA (Rahman et al., 2004). Interestingly, these effects could be direct, i.e., mediated by nAChRs expressed in DA neurons, or indirect, via α6^∗^nAChRs located pre-synaptically in mesencephalic GABAergic neurons projecting to DA neurons (Yang et al., 2011). Since both mouse and human cultures used in the present article contained about 20–25% of GABAergic neurons (Collo et al., 2018), it cannot be excluded that the nicotine-induced structural plasticity of DA neurons could be ascribed, at least in part, to a reduced GABAergic inhibitory drive on the other neurons present in our cultures, including the DA neurons, produced by sub-chronic exposure to nicotine.

Further support to this interpretation was offered by the effects of D3 receptor (D3R)-preferring D2/D3 agonists, such as 7-OHDPAT and ropinirole, and indirect agonists that increase extracellular DA levels, such as amphetamine or cocaine. Accordingly, all these agents engage the intracellular Ras-ERK1/2 and the PI3K-Akt-mTOR pathways via D3R-Gi signaling, resulting in increase of soma size and dendrite arborization when assessed 3 days after exposure (Collo et al., 2012, 2018). These effects were blocked by pretreatments with the selective D3R antagonists, such as SB277011-A (Collo et al., 2012, 2018). Interestingly, these D3R antagonists were also able to block the structural plasticity produced by nicotine on mouse DA neurons (Collo et al., 2013), suggesting a permissive and necessary role of D3R-dependent DA neurotransmission to allow the full expression of the structural plasticity effects of nicotine.

In the present study in addition to α-CTX MII and α-CTX PIA, we also observed that also DHβE and mecamylamine blocked nicotine-induced structural plasticity in human DA neurons, as previously shown in mice (Collo et al., 2013). These data, when considered together, strongly indicate α4β2^∗^, α6β2^∗^, and α4α6β2^∗^nAChRs mediate nicotine-induced structural plasticity in both mouse and human DA neurons. This induced structural plasticity in DA neurons can represent a mechanism that critically contributes to the compulsive aspects of nicotine dependence in mice and, possibly, also in humans. In fact, the same α4β2^∗^, α6β2^∗^, and α4α6β2^∗^nAChRs have been consistently associated to various aspects of the sensitizing and reinforcing effects of nicotine in mice, including increased DA release (Maskos et al., 2005; Drenan et al., 2010; Exley et al., 2011). The human relevance of these translational observations is further supported by the fact that varenicline, an effective treatment indicated for nicotine dependence, was originally developed as partial agonist for α4β2^∗^nAChR and only recently found to be a potent partial agonist also on α6β2^∗^nAChR (Bordia et al., 2012).

The key role of α6^∗^nAChRs in controlling DA-mediated behavior was recently confirmed in transgenic mice expressing a gain-of-function 6Ld mutation in the α6 gene CHRNA6 (Drenan et al., 2008). Constitutive expression of the 6Ld mutation conferred exaggerated behavioral responses to nicotine, high liability to addiction, increased DA release to a nicotine challenge, and induction of functional plasticity phenomena, such an abnormal increase of GluR1 AMPA receptor subunit expression in the VTA DA neurons (Drenan et al., 2010; Berry et al., 2015). Genetic association for the risk of developing nicotine dependence was recently confirmed for one polymorphism within the CHRNB3–CHRNA6 gene cluster (Wen et al., 2016). Hypothetically, this liability may become particularly relevant in case an individual carrying some nAChR risk polymorphisms is exposed to nicotine during the last gestational period, as in the case of smoking women during pregnancy. Large observational studies indicate that individuals exposed to prenatal cigarette smoke show an increased risk to develop nicotine dependence and behavioral disturbances as adolescents (Goldschmidt et al., 2012; De Genna et al., 2017). Therefore, using our paradigm that consists of neurons with phenotypes typical of an early stage of postnatal development in mouse and human, the former from dissection of embryo mesencephalon and the latter from iPSC-derived preparations (Quadrato et al., 2016; Ardhanareeswaran et al., 2017), we may have the possibility to explore some aspects of the response to pharmacological agents in the early stages of mammalian life development.

Intriguingly, in the present work, we showed that prenatal exposure to nicotine in mice embryos from day E12.5 till E17.5 produced structural plasticity in the VTA/SN DA neurons at postnatal day 1. Prenatal exposures to nicotine during a similar embryo life period resulted in impaired behavior of the mice when assessed as young adults (Alkam et al., 2013, 2017). Here, we observed an increase of DA neuron soma size in both VTA and SN of newborn mice at postnatal day 1. This effect was dependent upon α6^∗^nAChR and α4α6^∗^nAChR since was lacking in α6KO and α4α6KO mice, respectively. Interestingly, prenatal exposure to nicotine was also shown to produce α4β2nAChR-dependent maladaptive neuroplasticity, with an excessive dendritic and spine outgrowth in the cortex of wild-type adolescent offspring mice, an effect that was shown to be calcium-signaling dependent (Jung et al., 2016).

The concept of maladaptive neuroplasticity induced by addictive drugs, such as amphetamine, cocaine, or nicotine, was originally proposed on the basis of findings obtained in rodents (Robinson and Kolb, 2004; Russo et al., 2010), with a shared conceptual relevance also for humans, unfortunately supported by a limited number of findings. It is tempting to suggest that the present study, showing similar maladaptive structural plasticity occurring *in vitro* for both mouse and human DA neurons, is adding translational relevance, at least at the cellular level. Support for the use of iPSC-derived DA neurons in assessing certain drug-induced molecular mechanisms was recently provided (Cooper et al., 2012; Avior et al., 2016). Regarding nicotine effects, human iPSC-derived DA neurons were studied from donors with the α5D398N polymorphism in the α5 subunit gene (CHRNA5) to assess the functional effects of such mutation (Deflorio et al., 2017). Since α5D398N was associated in genome-wide association studies (GWAS) with high risk to develop nicotine dependence, a differential sensitivity to nicotine was expected. Indeed, DA neurons from subjects carrying the α5D398N polymorphisms showed less sensitivity to acetylcholine and nicotine as measured as electrophysiological response using *in vitro* patch-clamp.

It is tempting to suggest that this evidence of a genetic risk, the critical role of α6^∗^nAChRs on DA neurons in the reward circuit and the identification of α6^∗^nAChRs-selective compounds are all pointing to a possible treatment for various type of substance dependence, including tobacco, psychostimulants, and alcohol, as proposed (Jackson et al., 2009; Quik et al., 2011).

## Conclusion

In this work, we provided evidence that human DA neurons differentiated from iPSCs from healthy donors offer a useful translational *in vitro* model for biological and pharmacological mechanisms initially assessed in primary culture from mouse embryos. We showed that nicotine induced structural plasticity in mouse and human DA neurons may represent critical maladaptive features of the midbrain DA system occurring in nicotine dependence, in particular, during development in individuals exposed to nicotine during gestational period, as in case of a pregnant smoker mother. We also identified a critical role of α6^∗^nAChR and probably α4α6β2^∗^nAChR in mediating the structural plasticity effects of nicotine, suggesting their possible relevance as a potential target for early therapeutic interventions.

## Ethics Statement

Human iPSCs were generated in Collo et al. (2018) Neural Plasticity, February 4, 2018:4196961 in accordance with the recommendations and following the approval of the local Ethics Committee (CEIOC – Fatebenefratelli Hospital “San Giovanni di Dio” – Brescia, Italy, 44/2001 and 39/2005). All subjects gave written informed consent for use in research applications. No human subjects were involved in the present study.

## Author Contributions

GC, EMP, and MZ participated in the research design. LC and GC conducted the experiments. UM provided the KO mice. GC and EMP performed the data analysis. GC, EMP, MZ, LC, UM, and ER contributed to the writing of the manuscript.

## Conflict of Interest Statement

The authors declare that the research was conducted in the absence of any commercial or financial relationships that could be construed as a potential conflict of interest.
