# Supplementary material for: Alpha6-Containing Nicotinic Acetylcholine Receptors Mediate Nicotine-Induced Structural Plasticity in Mouse and Human iPSC-Derived Dopaminergic Neurons
Source: Front Pharmacol. 2018 Jun 1;9:572. doi: 10.3389/fphar.2018.00572 (PMC5992464; doi:10.3389/fphar.2018.00572)
Supplement: Supplementary file 1 [file Image_1.pdf]

Supplementary Materials and Methods

Alpha6-containing Nicotinic Acetylcholine Receptors Mediate Nicotine-induced Structural Plasticity in Mouse and Human iPSC-derived Dopaminergic Neurons

Ginetta Collo, Laura Cavalleri, Michele Zoli,

Uwe Maskos, Emiliangelo Ratti, Emilio Merlo Pich

Supplementary Table S1. Pharmacological agents used in this study.

Name	Supplier	Catalog number	Solvent	Final concentration
(-)-Nicotine ditartrate	Tocris Bioscience	3546	Water	0.1-10 µM
BDNF	Alomone Labs	B-250	Water	10 ng/ml
α-conotoxin MII	Tocris Bioscience	1340	Water	10-100 nM
α-conotoxin PIA	Tocris Bioscience	3121	Water	100 nM
Mecamylamine	Tocris Bioscience	2843	Water	100 µM
Dihydro-β-erytroidine	Tocris Bioscience	2349	Water	10 µM
Methyllycaconitine	Tocris Bioscience	1029	Water	200 nM

Supplementary Table S2. Primary antibodies used in this study.

Name	Supplier	Catalog number	Species	Final dilution	Detection method	RRID
DAT	Santa Cruz Biotechnology	sc-32258	Rat mAb	1:400	IF	AB_627400
MAP2	Merck Millipore	AB5622	Rabbit pAb	1:1000	IF	AB_91939
TH	Merck Millipore	MAB318	Mouse mAb	1:500	IF	AB_2201528
TH	Santa Cruz Biotechnology	sc-14007	Rabbit pAb	1:500	IF, ICC	AB_671397

Supplementary Table S3. Secondary antibodies used in this study.

Name	Supplier	Catalog number	Final dilution	Detection method	RRID
Biotinylated goat anti-rabbit IgG	Vector Laboratories	BA-1000	1:350	ICC	AB_2313606
Cy™3-conjugated goat anti-mouse IgG	Jackson ImmunoResearch	115-166-062	1:800	IF	AB_2338703
Cy™3-conjugated goat anti-rat IgG	Jackson ImmunoResearch	112-165-167	1:1000	IF	AB_2338251
Alexa Fluor® 488-conjugated goat anti-rabbit IgG	Jackson ImmunoResearch	111-485-144	1:500	IF	

Supplementary Table S4. Media and reagents.

Name	Supplier	Catalog number
2-mercaptoethanol	Gibco	21985023
ABComplex	Vector Laboratories	PK-6100
Accumax	Sigma-Aldrich	A7089
Accutase	StemCell Technologies	07920
Ascorbic acid	Sigma-Aldrich	A4403
B27 supplement	Gibco	17504044
BDNF	R&D Systems	248-BD
BSA	Sigma-Aldrich	A2153
CHIR99021	Stemgent	04-0004
DAB	Sigma-Aldrich	D5637
DAPI	Molecular Probes	D1306
DAPT	Tocris Bioscience	2634
Dibutyryl cAMP	Sigma-Aldrich	D0627
FGF8	R&D Systems	423-F8
Fibronectin	Sigma-Aldrich	F4759
GDNF	R&D Systems	212-GD
GlutaMAX™	Gibco	35050061
Knockout Serum Replacement	Gibco	10828028
Knockout™ DMEM	Gibco	10829018
Knockout™ DMEM/F12	Gibco	11330032
Laminin	Sigma-Aldrich	L2020
LDN193189	Stemgent	04-0074
L-Glutamine	EuroClone	ECB3000D
Matrigel	Corning	354234
N2 supplement	Gibco	17502048
Neurobasal medium	Gibco	21103049
Normal goat serum	EuroClone	BK5425S
Paraformaldehyde	Sigma-Aldrich	P6148
PBS	EuroClone	ECB4053L
Poly-DL-Ornithine	Sigma-Aldrich	P0671
Poly-D-Lysine	Sigma-Aldrich	P7405

Purmorphamine	Stemgent	04-0009
SB431542	Tocris Bioscience	1614
Shh C25II	R&D Systems	464-SH
Sucrose	Sigma-Aldrich	S7903
TGFβ3	R&D Systems	243-B3
Triton	Sigma-Aldrich	T8787

Supplementary Figure legends

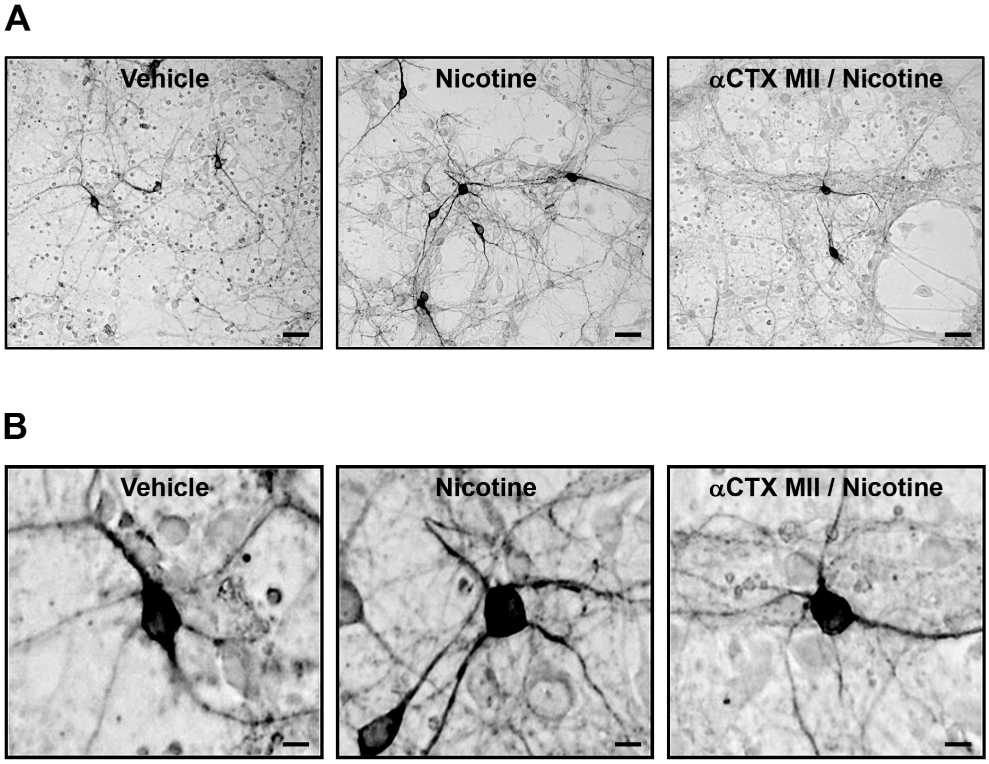

Supplementary Figure S1. *Structural plasticity induced by nicotine in mouse DA neurons and blockade with α-conotoxin MII.* **(A)** Low magnification photomicrographs of mouse mesencephalic DA neurons 72 hrs after exposure to vehicle, 10 μM nicotine or 100 nM α-conotoxin MII followed by nicotine (Scale bar: 50 μm). **(B)** High magnification photomicrographs of mouse mesencephalic DA neurons shown in panel (A) (Scale bar: 10 μm). Abbreviation: αCTX MII: α-conotoxin MII.

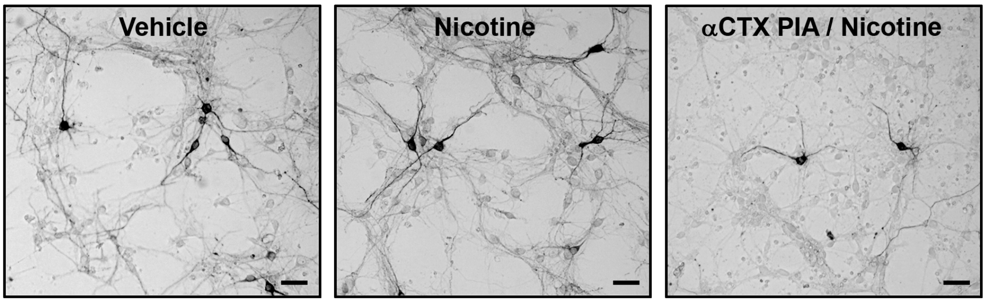

Supplementary Figure S2. *Blockade of nicotine-induced structural plasticity by* α-*conotoxin PIA.* Low magnification photomicrographs of mouse mesencephalic DA neurons 72 hrs after exposure to vehicle, 10 μM nicotine or 100 nM α-conotoxin PIA followed by nicotine (Scale bar: 50 μm). Abbreviation: αCTX PIA: α-conotoxin PIA.

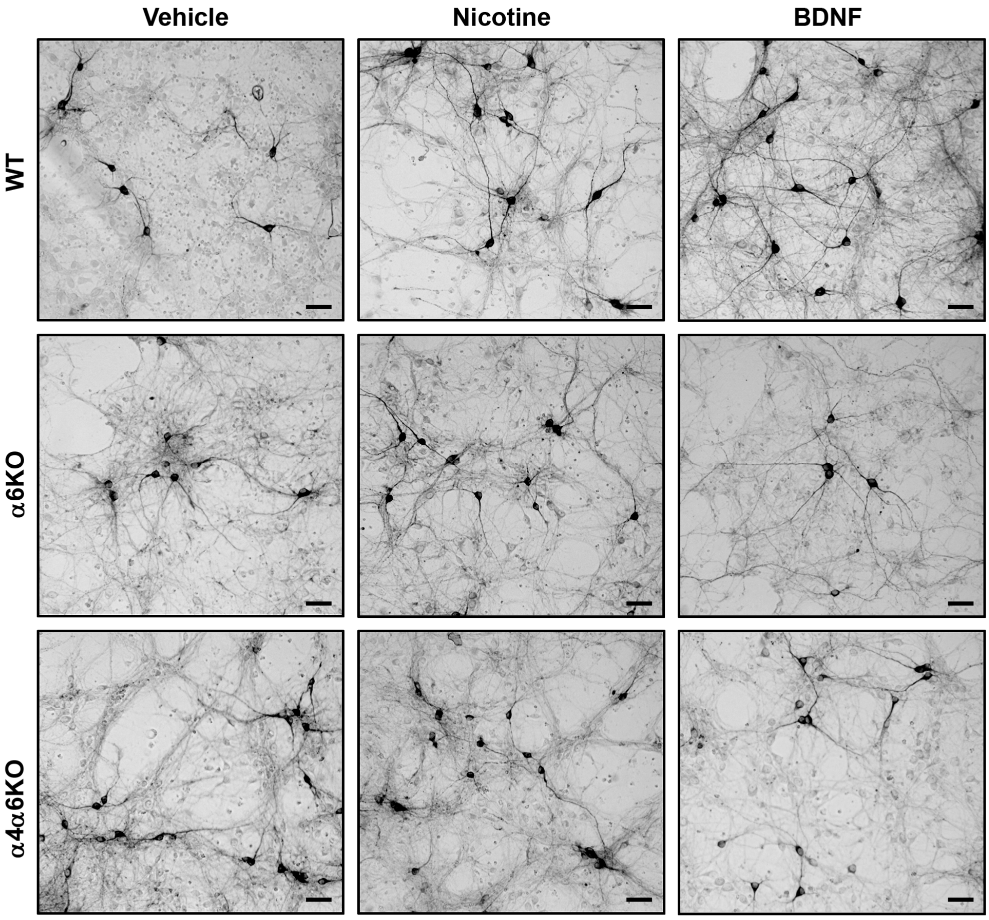

Supplementary Figure S3. *Structural plasticity induced by nicotine in mesencephalic DA neurons from wild-type and α6 or α4/α6 nAChR subunit null mutant mice.* Low magnification photomicrographs of mouse mesencephalic DA neurons from wild-type, α6KO and α4/α6KO mice 72 hrs after exposure to vehicle, 10 μM nicotine or 10 ng/ml BDNF (Scale bar: 50 μm). Abbreviation: WT: wild-type; BDNF: brain derived neurotrophic factor.

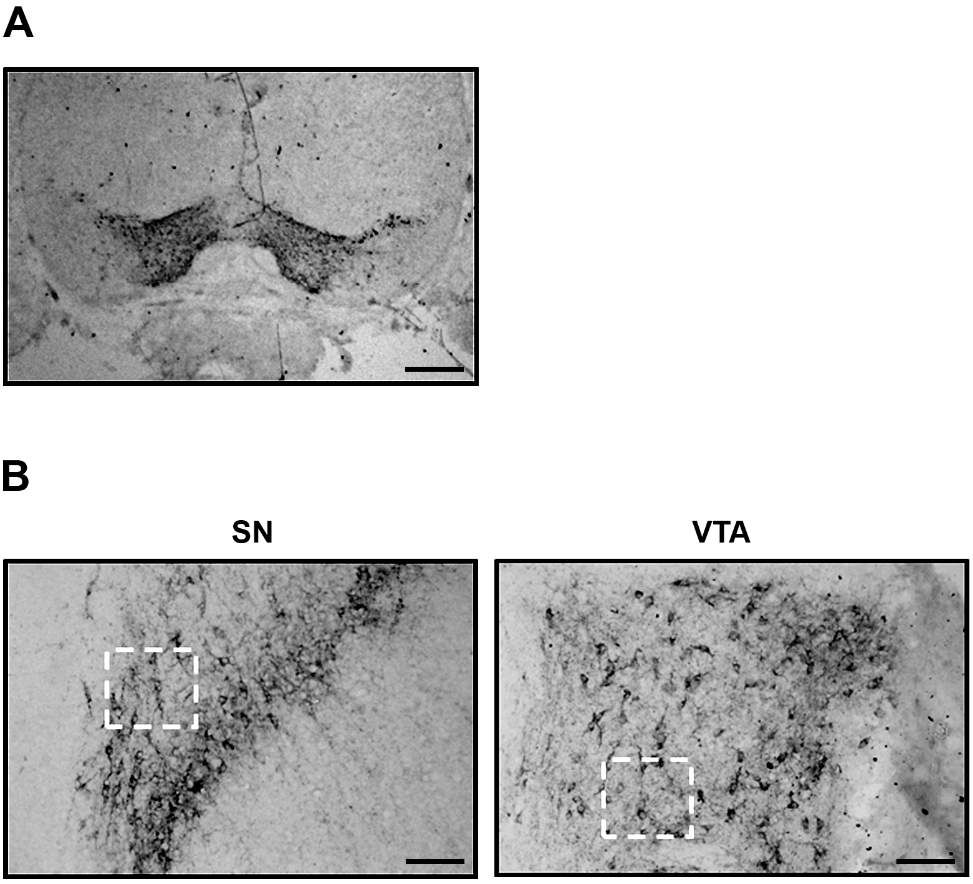

Supplementary Figure S4. *Photomicrographs of SN and VTA of P1 mice.* Representative low magnification photomicrographs of midbrain DA neurons from **(A)** a coronal brain section of a P1 mouse and **(B)** SN and VTA. The white drawing on the two images indicates the areas showed at high magnification in Figure 4. (Scale bar: (A) = 500 μm; (B) = 100 μm). Abbreviations: SN: substantia nigra; VTA: ventral tegmental area.

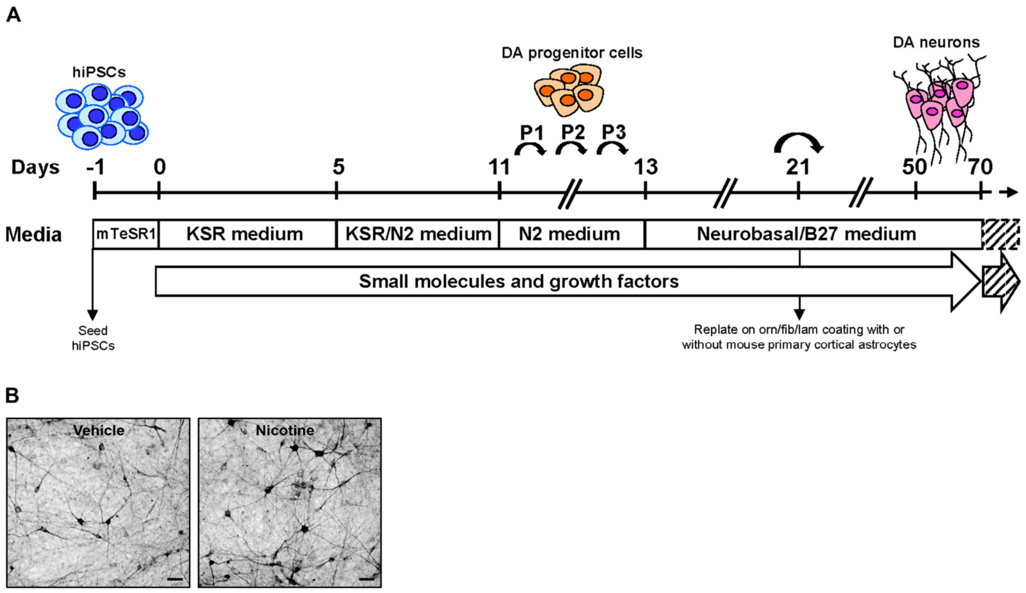

Supplementary Figure S5. *Differentiation of human iPSCs into DA neurons and response to nicotine.* **(A)** Diagram showing time and conditions used for the differentiation of DA neurons from human iPSCs. **(B)** Low magnification photomicrographs of human DA neurons at 70 days in culture, assessed at 72 hrs after exposure to vehicle or 10 µM nicotine (Scale bar: 50 µm).

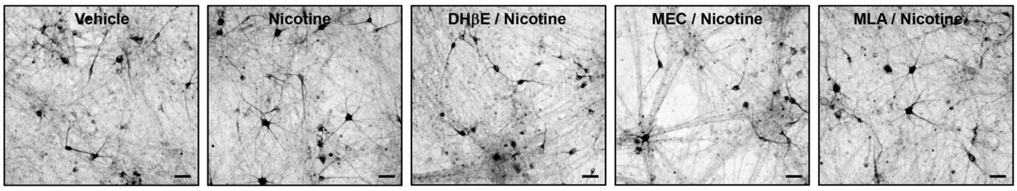

Supplementary Figure S6. *Blockade of nicotine-induced structural plasticity in DA neurons differentiated from human iPSCs by nAChR antagonists.* Low magnification photomicrographs of human DA neurons at 70 days in culture, assessed at 72 hrs after exposure to vehicle, 10 μM nicotine or pretreatments with dihydro-β-erythroidine (10 μM), mecamylamine (100 μM) and methyllycaconitine (200 nM) followed by nicotine (Scale bar: 50 μm). Abbreviations: DHβE: dihydro-β-erythroidine; MEC: mecamylamine; MLA: methyllycaconitine.

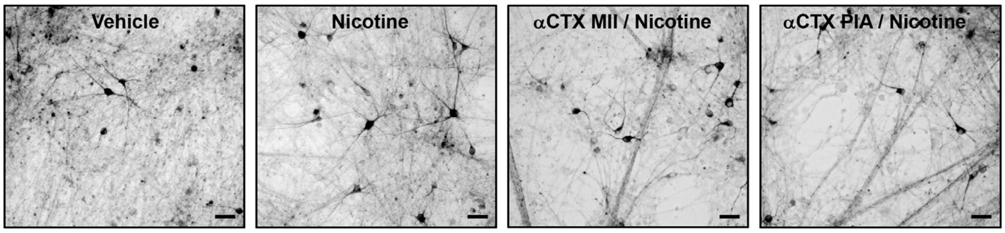

Supplementary Figure S7. *Blockade of nicotine-induced structural plasticity in DA neurons differentiated from human iPSCs by α-conotoxin MII and α-conotoxin PIA.* Low magnification photomicrographs of human DA neurons at 70 days in culture, assessed at 72 hrs after exposure to vehicle, 10 μM nicotine or pretreatments with α-conotoxin MII (100 nM) and α-conotoxin PIA (100 nM) followed by nicotine (Scale bar: 50 μm). Abbreviations: αCTX MII: α-conotoxin MII; αCTX PIA: α-conotoxin PIA.
